# Diagnostic Utility of Hair Toxic Metals in Autism Spectrum Disorder: Unlocking Biomarkers Among Egyptian Children

**DOI:** 10.1007/s12011-025-04877-7

**Published:** 2025-11-10

**Authors:** Ammal M. Metwally, Tarek M. Y. Omar, Ehab R. Abdel Raouf, Engy A. Ashaat, Amal Elsaeid, Mostafa M. El-Saied, Asmaa E. Elkhouly, Naglaa Abu-Mandil Hassan, Mona A. Helmy, Amira A. Goda, Dina Abu Zeid, Amira S. ElRifay, Sherif E. ElDeeb, Ghada A. Elshaarawy, Nevin E. Sharaf

**Affiliations:** 1 Community Medicine Research Department, Medical Research and Clinical Studies Institute, National Research Centre (Affiliation ID, Dokki, Cairo, 60014618) Egypt; 2 Department of Pollution, Agricultural Research Center, Giza, Egypt; 3 Child With Special Needs Department, Medical Research and Clinical Studies Institute, National Research Centre (Affiliation ID, 60014618) Dokki, Cairo, Egypt; 4 Clinical Genetics Department, Human Genetics and Genome Research Institute, National Research Centre (Affiliation ID, Dokki, Cairo, 60014618) Egypt; 5 Medical Molecular Genetics Department, Human Genetics and Genome Research Division, National Research Centre (Affiliation ID, 60014618) Dokki, Cairo, Egypt; 6 Biological Anthropology Department/Medical Researchand , Clinical Studies Institute/National Research Centre (Affiliation ID, Dokki, Cairo, 60014618) Egypt; 7 Environmental and Occupational Medicine Department, Environmental and Climate Change Research Institute, National Research Centre (Affiliation ID, Dokki, Cairo, 60014618) Egypt; 8 Department of Food Contaminants and Toxicology/Food Industryand , Nutrition Research Institute/National Research Centre (Affiliation ID, Dokki, Cairo, 60014618) Egypt; 9 Child Health Department, Medical Research and Clinical Studies Institute, National Research Centre (Affiliation ID, 60014618) Dokki, Cairo, Egypt

**Keywords:** Autism Spectrum Disorder (ASD), Toxic Metals, Hair Biomarkers, Diagnostic Cutoff Values, Aluminum, Lead

## Abstract

This study aimed to assess the diagnostic utility of hair-based toxic metal profiling in Egyptian children with autism spectrum disorder (ASD). The objectives included establishing population-specific cutoff values for selected metals and examining their correlation with ASD symptom and severity. A national, facility-based comparative study was conducted across eight Egyptian governorates. It included 455 children with clinically confirmed ASD and 230 age- and sex-matched typically developing relatives. ASD diagnoses were established using DSM-5 criteria and the Gilliam Autism Rating Scale, Second Edition (GARS-2). Hair samples were collected and analyzed for five toxic metals; aluminum (Al), arsenic (As), cadmium (Cd), mercury (Hg), and lead (Pb) using inductively coupled plasma mass spectrometry (ICP-MS). Receiver operating characteristic (ROC) curve analysis was applied to determine optimal diagnostic cutoffs. Sensitivity, specificity, and predictive values were calculated. Correlation analyses were conducted to evaluate the association between metal levels and ASD severity. Children with ASD had significantly elevated hair levels of Al, As, Cd, and Pb compared to typically developing controls. Hg levels were statistically insignificant higher in the ASD group. Al showed the strongest diagnostic performance with 89.4% sensitivity and a cutoff ≥ 10.35 ppm. Proposed cutoff values for the other metals were: As ≥ 0.10 ppm, Cd ≥ 0.10 ppm, Hg ≥ 0.31 ppm, and Pb ≥ 1.87 ppm. Only Al levels were positively correlated with ASD severity. Hair-based toxic metal profiling, particularly of Al, may support early ASD risk detection. However, it should be integrated within a broader diagnostic framework that includes clinical, behavioral, and environmental assessments.

## Introduction

Autism spectrum disorder is a complex neurodevelopmental condition characterized by persistent difficulties in social communication and interaction, accompanied by restricted, repetitive behaviors and interests. These symptoms typically emerge in early childhood and can lead to lifelong functional challenges if not diagnosed and addressed early [1]. The condition was first described by Leo Kanner in 1943 as a distinct clinical entity characterized by early-onset social and communicative deficits and repetitive behavioral patterns [2].

Globally, the prevalence of ASD has increased dramatically in recent decades, now affecting approximately 1 in 36 children, according to recent surveillance reports [3]. In Egypt, a nationally representative survey revealed that 3.3% of children aged 1 to 12 years are at high risk of ASD [4]. Egyptian children confirmed nationally with ASD amounted to 1.1% [5], emphasizing the urgency of enhancing early identification and intervention strategies [4].

While ASD is widely recognized as a condition influenced by genetic and epigenetic factors [6–8], mounting evidence points to the significant role of environmental exposures, particularly toxic metals in its etiology [9, 10]. Although the term “heavy metals” is frequently used in the literature, it is scientifically imprecise and often misleading [11, 12]. In the present study, we adopt the more accurate term “toxic metals”, since all five investigated elements (Al, Pb, Hg, As, Cd) are well-established in the literature as toxic in various forms, depending on dose and chemical speciation [13, 14].

Metals such as Al, Pb, Hg, As, and Cd have been implicated in neurodevelopmental disruption through their capacity to induce oxidative stress, impair antioxidant systems, and alter gene expression [15, 16]. Elevated levels of these metals have frequently been detected in children with ASD, suggesting a potential role as early biological indicators [9, 17]. However, the lack of standardized diagnostic thresholds, inconsistent methodologies across studies, and population-specific exposure differences (e.g., industrial pollution, nutrition, cookware materials) have limited the clinical utility of toxic metal measurements [6, 7, 18, 19].

Moreover, previous studies have often reported inconsistent correlations between metal levels and ASD symptom severity, and few have applied robust diagnostic tools (e.g., ROC curves, AUC metrics) to evaluate their predictive value. This is especially critical in settings like Egypt, where environmental contamination poses a disproportionate burden, but localized reference values and diagnostic criteria remain poorly defined [20, 21].

This study evaluated the diagnostic utility of five toxic metals (Al, Pb, Hg, As, Cd) by analyzing their concentrations in the hair of Egyptian children with ASD compared to typically developing controls. It sought to establish Egyptian-specific cutoff values for each metal and assess their diagnostic accuracy using receiver ROC analyses, sensitivity, specificity, and predictive values. The study also explored how toxic metal burden relates to ASD symptom severity, employing both correlation and quartile-based analyses to identify dose–response trends, particularly for aluminum. In addition, regional and demographic variations, including sex, residence, and degree of industrialization, were analyzed to uncover environmental patterns influencing exposure risk. Collectively, these analyses provide population-specific insights into the contribution of toxic metal exposure to ASD pathophysiology and inform the development of evidence-based, environmentally tailored screening strategies for early ASD risk detection.

## Methods

### Study Design and Setting:

This study was part of a broader national project entitled "National Prevalence Survey for ASDs: Assessing its Epidemiological Pattern and Risk Factors." It combined both community-based and facility-based approaches and was implemented across four years (December 2017 to December 2021) in eight governorates representing Egypt's major geographic regions. The selected governorates included urban (Cairo), rural, Upper Egypt (Fayoum, Assiut, and Aswan), Lower Egypt (Damietta, Dakahlia, and Gharbia), and a Frontier region (Marsa Matrouh), providing comprehensive geographic and sociodemographic diversity.

The current manuscript focuses on a nested case–control diagnostic sub-study conducted within this national framework. This sub-study specifically aimed to evaluate the diagnostic utility of toxic metals in the hair of children with confirmed ASD versus matched healthy relatives.

### Study Phases and Participant Recruitment

The overall national study was conducted in four distinct phases. Phase I involved community-based household screening of 41,640 children (ages 1–12) using the Arabic version of the Vineland Adaptive Behavior Scales (VABSA) [22]. Children scoring < 85 were referred for further evaluation. In Phase II, high-risk children were screened in maternal and child health facilities using the Modified Checklist for Autism in Toddlers (M-CHAT) for ages 1–3 [23] and the Gilliam Autism Rating Scale-2 (GARS-2) for ages 3–12 [24]**,** supplemented by the Denver II Developmental Screening Test [25]. Phase III involved confirmatory diagnosis by neurodevelopmental specialists using DSM-5 criteria [26] and CARS [27]. Phase IV addressed risk factor assessment, assessment of toxic metals under study in the hair of the participants enrolled.

### Participants

Children with confirmed ASD diagnoses were invited to participate in the current diagnostic sub-study, along with age- and sex-matched healthy relatives who served as typically developing controls.

#### Inclusion criteria

Children aged 1–12 years who met DSM-5 diagnostic criteria for ASD [26], either previously diagnosed or newly confirmed during the study, were eligible. Matched typically developing controls included healthy relatives in the same age range, with no history of neurodevelopmental disorders.

#### Exclusion criteria 

Children with known or previously diagnosed genetic disorders (e.g., Down syndrome, Turner syndrome, fragile-X syndrome), those with hearing or visual impairments, significant orthopedic conditions, or developmental disabilities unrelated to ASD were excluded. Verification of genetic or medical conditions was sought through clinical geneticist evaluation or supporting documentation. All assessments were conducted using validated Arabic versions of screening tools [22, 28, 29].

Control participants were age- and sex-matched relatives (siblings or cousins) of ASD cases. This approach minimized socioeconomic and regional heterogeneity, which are major confounders in environmental studies. However, it may have partially attenuated between-group contrasts due to shared household and dietary exposures. A complementary study by our team (currently under publication) focuses specifically on environmental and behavioral factors within this cohort to further characterize exposure determinants.

### Rationale for Age Range (1–12 years)

This age range was selected based on variability in the onset and recognition of ASD symptoms. Early signs may emerge between 12–24 months, but formal diagnoses are often confirmed later. The American Academy of Pediatrics recommends screening at 18 and 24 months, but many children are diagnosed at school age [30]. Therefore, the enrolled participants covered a wide range of age to capture children for early detection, established diagnosis, and later or overlooked children with ASD.

### Sampling and Cluster Selection

A multistage, stratified cluster sampling strategy was used. In Stage 1, eight governorates were purposively selected to represent geographic and socioeconomic diversity based on CAPMAS 2017 census data [31]. In Stage 2, urban cities (kism) and rural villages (markaz) were selected according to human development index categories [32, 33]. In Stage 3, one urban and one rural cluster from each category were randomly selected, resulting in 45 clusters (24 urban, 21 rural). The national sample included 41,640 children, yielding 455 children with confirmed ASD and 230 matched healthy relatives for the current study [5].

### Sample Size Determination

A target sample size of 460 children with ASD and 230 typically developing controls was calculated to evaluate the diagnostic performance of toxic metals with 95% statistical power [34–36]. The calculation assumed a baseline proportion of 0.50 in the ASD group under the null hypothesis, rising to 0.65 under the alternative hypothesis, with a corresponding control group proportion of 0.50. A two-sided Z-test was used at a 0.05 significance level. Anticipating incomplete matching between children with ASD and relatives, along with an expected 10% attrition, the finalized sample included 455 children with ASD, compromising a high response rate of 98.9%, and 230 matched typically developing controls. This configuration was sufficient to detect a 15% difference in proportions with the desired statistical power.

Based on these calculations and the observed data distribution, the power of this study to detect significant differences in toxic metal concentrations between ASD and control groups exceeded 95%. This level of statistical power provides confidence in the robustness and reliability of the diagnostic performance outcomes.

For the nested diagnostic performance analysis, this sample size yielded robust power to detect group differences in metal concentrations and to establish diagnostic cutoff values using ROC curve analysis.

### Hair Sample Collection and Toxic Metal Analysis

Hair samples were selected as the biological matrix for toxic metal assessment due to their practicality and scientific reliability in pediatric studies. Unlike blood or urine, which mainly reflect recent exposure, hair provides a long-term record of metal accumulation, representing exposure over several months up to a year. In addition, hair sampling is simple, painless, and more acceptable to children with ASD, in whom venipuncture or urine collection can be challenging. This approach has been supported by previous research demonstrating that hair concentrations of metals reliably reflect chronic exposure patterns among children with autism [37].

Hair samples (~ 0.1 g) were cut from the nape of the neck, a site less prone to environmental contamination. To further minimize the risk of external interference; such as cosmetic products, dust, or environmental pollutants—samples underwent a stringent decontamination protocol. Each sample was initially washed using a non-ionic detergent to remove surface residues, followed by multiple rinses with deionized water, then air-dried in a clean environment.

Following washing and drying, hair samples were finely cut and ground into uniform segments (~ 1–2 mm) using a clean stainless-steel micro-homogenizer to ensure representative digestion. Approximately 0.1 g of the homogenized hair was digested in a closed-vessel microwave system using ultra-pure nitric acid (HNO₃, 65%) and hydrogen peroxide (H₂O₂, 30%) at 180 °C until complete mineralization. The digest was then diluted with ultrapure deionized water and analyzed for Al, As, Cd, Hg, and Pb using inductively coupled plasma triple quadrupole mass spectrometry (ICP-QQQ; Agilent 8800). Calibration curves were generated using certified multi-element standards, and quality control was maintained through reagent blanks, spike recovery tests, and certified reference materials (NCS DC73347, human hair). All procedures followed international quality standards and previously validated methods [37].

The cleaned samples were digested using nitric and perchloric acid under controlled laboratory conditions. Toxic metals (Al, As, Cd, Hg, Pb) were quantified using inductively coupled plasma triple quadrupole mass spectrometry (ICP-QQQ, Agilent 8800). Instrument calibration was performed using certified internal standards and matrix-matched spike solutions. To ensure accuracy and reproducibility, each batch included reagent blanks and certified reference materials. All procedures complied with international quality assurance standards [18, 38].

### ASD Severity and Behavioral Assessment

Autism severity was classified using DSM-5 levels and quantified via the Childhood Autism Rating Scale (CARS), which has excellent internal consistency (Cronbach’s alpha = 0.82–0.95) and validated Arabic versions [27]. According to the CARS manual, ASD is defined as a CARS score of ≥ 30 points. CARS scores between 30 and 36.5 indicate mild to moderate autism, while scores between 37 and 60 suggest severe autism.

### Statistical Analysis and Diagnostic Evaluation

Data were coded and analyzed using IBM SPSS (version 22.0). Normality was checked with the Shapiro–Wilk test, with normally distributed data expressed as mean ± SD. The t-test compared continuous variables; the chi-squared test and Fissure exact test compared categorical data.

For stratified analysis, governorates were grouped into two broad categories according to their industrial and environmental context: Industrialized/Cities/Delta (Cairo, Dakahlia, Damietta, Gharbia) and Non-Industrialized/Upper Egypt/Frontier (Fayoum, Assiut, Aswan, Marsa Matrouh). This classification reflects the well-documented environmental divide in Egypt. The Nile Delta and Greater Cairo are characterized by high population density, heavy traffic, industrial clusters (cement, textile, chemical), and intensive agricultural practices, all of which contribute to elevated levels of toxic metal emissions and residues [1, 3, 6, 39–41]. In contrast, Upper Egypt and frontier governorates are less industrialized, more rural or desert-based, and host fewer major industrial complexes, resulting in lower background contamination levels [7, 42] This binary stratification has been used in environmental monitoring reports and provides a meaningful framework to examine how background exposure influences toxic metal concentrations in ASD and control children.

Statistical analysis was extended to include two-way ANOVA of log10-transformed hair toxic metal concentrations across governorates among children with ASD and controls separately and (Group × Governorate) to evaluate interaction effects. Effect sizes were reported as η^2^ for ANOVA.

Diagnostic performance metrics for each toxic metal cutoff included: Sensitivity (true positive rate), Specificity (true negative rate), Positive/Negative Predictive Values (PPV/NPV)**,** Diagnostic Accuracy**,** Youden’s Index (sensitivity + specificity – 1), and Likelihood Ratios (LR + and LR–) and area under the curve (AUC) for ROC analyses, with significance set at *p* < 0.05.

## Results

### Demographic characteristics of the study population

The study enrolled a total of 455 children with ASD and 230 age- and sex-matched typically developing controls, all drawn from a nationally representative sample across eight Egyptian governorates. The mean age of children in the ASD group was 6.7 ± 2.9 years*,* while that of the control group was 6.5 ± 2.6 years; this difference was not statistically significant (p = 0.21*),* indicating a well-balanced age distribution between the two groups. Boys predominated in both groups, comprising 73.2% of the ASD cohort and 70.9% of the typically developing controls, reflecting the higher prevalence of ASD typically observed in boys. Children with ASD and typically developing controls were comparable in locality and geographical distribution (*p* > 0.05).This demographic balance strengthens the internal validity of the diagnostic comparison and helps to control for potential confounding effects related to age and sex (Table 1).

**Table 1:** Comparison of the epidemiological characteristics and mean hair concentrations (ppm) of toxic metals among children with ASD and typically developing controls

Demographic data	Children with ASD (Total = 455)	Typically developing controls (Total = 230)	p-value
**Age of children (years)#**	6.6 ± 2.5	6.5 ± 2.7	^0.555
**Gender:** Boys Girls	368 (80.9%) 87 (19.1%)	182 (79.1%) 48 (20.9%)	Ω 0.587
**Locality:** Urban Rural	305 (67.0%) 150 (33.0%)	140 (60.9%) 90 (39.1%)	Ω 0.110
**Geographical Distribution by industrialization** Industrialized Non-industrialized	254 (55.8%) 201 (44.2%)	126 (54.8%) 104 (45.2%)	Ω 0.796
**Toxic metals levels (ppm)#**			
Al	16.55 ± 6.26	9.94 ± 5.04	^ < 0.001**
As	0.23 ± 0.43	0.11 ± 0.10	^ < 0.001**
Cd	0.18 ± 0.18	0.13 ± 0.11	^0.002**
Hg	0.47 ± 0.43	0.39 ± 0.40	^0.104
Pb	1.79 ± 1.13	1.44 ± 0.74	^0.001**

#Data presented as Mean±SD. Test of significance: ^Independent t-test, Ω Chi Square, **highly significant at p value < 0.01

### Hair metal concentrations among ASD and control groups

Quantitative analysis of hair samples revealed significantly elevated concentrations of four toxic metals; Al, As, Cd, and Pb in children with ASD when compared to their healthy counterparts (*p *< 0.001 for all comparisons) as shown in Table 1. Hg concentrations, although higher in the ASD group, did not reach statistical significance (*p* = 0.07), suggesting a weaker discriminatory role in this population.

Specifically, the mean Al concentration in the ASD group was markedly higher (16.55 ± 7.84 ppm) than in typically developing children (9.94 ± 3.90 ppm). As and Cd, while present at lower absolute concentrations, also exhibited statistically significant differences between children with ASD and typically developing controls. The mean As levels were 0.23 ± 0.08 ppm in children with ASD versus 0.11 ± 0.06 ppm in typically developing controls, and Cd levels were 0.18 ± 0.07 ppm compared to 0.13 ± 0.05 ppm, respectively. Pb concentrations followed a similar pattern, for children with ASD; exhibiting mean levels of 1.79 ± 0.61 ppm compared to 1.44 ± 0.53 ppm in the control group. These findings collectively support the hypothesis that dysregulation of metal metabolism or environmental exposure may be associated with ASD etiology or symptomatology.

### Sociodemographic Determinants of Toxic Metal Burden

Sociodemographic determinants of toxic metal variations by gender (boys and girls) and residence (urban vs rural) between ASD and typically developing controls were shown in Table 2. Gender exerted minimal influence on metal levels; only Pb was significantly higher among ASD girls (2.10 ± 1.03 ppm) than boys (1.71 ± 1.14 ppm; *p* = 0.025). Residence showed a modest effect: rural ASD children had slightly higher Al levels (17.46 ± 6.90 ppm) than urban (15.94 ± 5.74 ppm), approaching significance (*p* = 0.055). Two-way ANOVA confirmed a residence effect for only Al (*p* = 0.032). The ANOVA confirms that ASD diagnosis is the dominant factor explaining toxic-metal variation, accounting for the largest share of between-group variance, especially for Al, As, and Pb**.**

**Table 2:** Sociodemographic determinants of hair toxic metal concentrations in children with ASD and typically developing controls

Variables	n (%)	Aluminum (Al)	Arsenic (As)	Cadmium (Cd)	Mercury (Hg)	Lead (Pb)
**Gender**						
**Children with ASD:** Boys Girls	455 368 (80.9%) 87 (19.1%)	16.41 ± 6.35 17.14 ± 5.95	0.25 ± 0.47 0.17 ± 0.19	0.18 ± 0.19 0.18 ± 0.16	0.48 ± 0.43 0.43 ± 0.40	1.71 ± 1.14 2.10 ± 1.03
*p value*^*#*^		0.458	0.203	0.991	0.412	0.025*
**T**ypically developing controls**:** Boys Girls	230 182 (79.1%) 48 (20.9%)	9.99 ± 5.26 9.71 ± 4.16	0.11 ± 0.11 0.12 ± 0.10	0.14 ± 0.12 0.10 ± 0.07	0.36 ± 0.36 0.39 ± 0.53	1.42 ± 0.79 1.52 ± 0.53
*p value*^*#*^		0.821	0.802	0.197	0.154	0.587
**Residence**						
**Children with ASD:** Urban Rural	455 305 (67.0%) 150 (33.0%)	15.46 ± 6.9017.94 ± 5.74	0.26 ± 0.53 0.19 ± 0.20	0.18 ± 0.20 0.17 ± 0.14	0.47 ± 0.45 0.47 ± 0.40	1.80 ± 1.07 1.77 ± 1.22
*p value*^*#*^		0.055	0.111	0.528	0.983	0.828
**T**ypically developing controls**:** Urban Rural	230 140 (60.9%) 90 (39.1%)	10.65 ± 5.60 8.95 ± 4.94	0.11 ± 0.11 0.10 ± 0.09	0.12 ± 0.14 0.14 ± 0.08	0.34 ± 0.31 0.38 ± 0.43	1.57 ± 0.71 1.34 ± 0.74
*p value*^*#*^		0.133	0.707	0.419	0.555	0.141
*p value^*		0.032 *	0.475	0.417	0.625	0.437
*p value*^*§*^		< 0.001**	< 0.001**	0.024 *	0.031 *	< 0.001 **

Tests of significant were: ^#^independent sample t test between means and ^§^two-way ANOVA between group (cases and controls), ^two-way ANOVA between group (Urban vs Rural), *significant at p value < 0.05, **highly significant at p value < 0.01.

### Geographic Variation in Hair Toxic Metal Concentrations

**Fig. 1:**
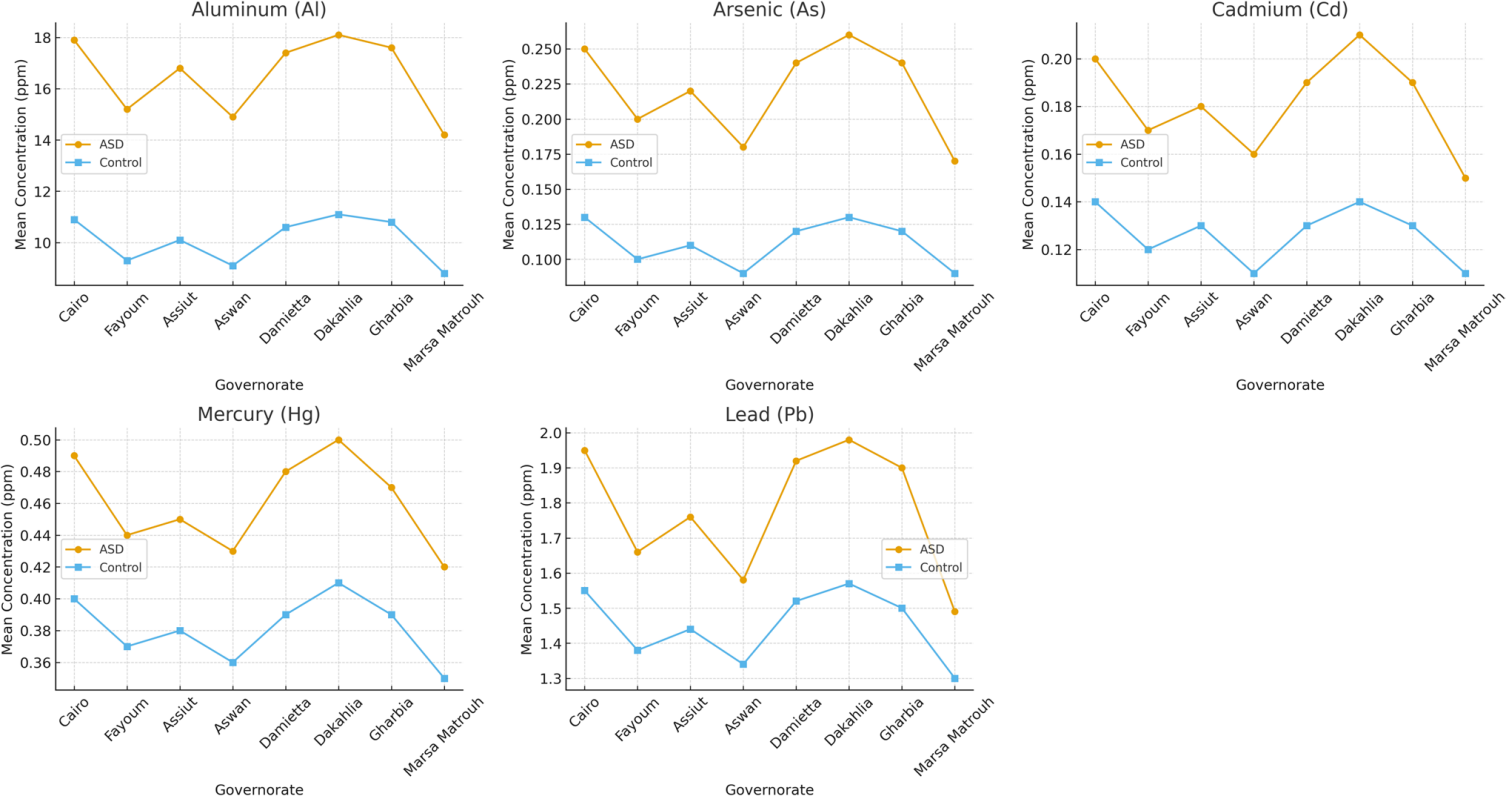
Geographic variation in hair toxic metal concentrations among children with ASD and typically developing controls

Geographic variations of hair toxic metal concentrations for children with ASD and typically developing controls were explored across governorates (Fig. 1) and per region (Table 3). Al and Hg showed the largest regional contrasts. Al, As, and Pb exhibited the most significant regional contrasts. Among children with ASD, Al concentrations were slightly higher in industrialized governorates (17.75 ± 7.0 ppm) compared with non-industrialized ones (15.88 ± 7.5 ppm). The same pattern was observed in typically developing controls (industrialized = 10.85 ± 3.9 ppm; non-industrialized = 9.53 ± 3.8 ppm). As followed a similar pattern, with elevated levels in children with ASD (0.25 ± 0.08 ppm) relative to typically developing controls (0.11 ± 0.06 ppm) across both regions. Hg and Cd showed smaller regional differences, while Pb concentrations were consistently higher among ASD children (1.94 ± 0.61 ppm) than controls (1.43 ± 0.53 ppm) in industrialized areas.

Two-way ANOVA confirmed significant group effects for all five metals (*p* < 0.05), as well as governorate effects for Al, As, Cd, Hg, and Pb (*p* < 0.01). Significant group and region interactions were identified for Al (*p* = 0.006) and Pb (*p* = 0.048), indicating that the influence of regional context on metal exposure differs between ASD and control groups.

Mean (± SD) concentrations of five toxic metals (Al, As, Cd, Hg, Pb) are shown across governorates for children ASD and typically developing controls. Data were log₁₀-transformed for analysis and back-transformed into ppm for display.

**Table 3:** Two-Way ANOVA of hair toxic metals levels (ppm) by group and region (Industrialized vs Non-Industrialized)

Region	Al Mean ± SD	As Mean ± SD	Cd Mean ± SD	Hg Mean ± SD	Pb Mean ± SD
**Industrialized (n = 380) §**					
**Children with ASD** (n = 254)	17.75 ± 7.0	0.25 ± 0.08	0.20 ± 0.07	0.49 ± 0.43	1.94 ± 0.61
**T**ypically developing controls (n = 126)	10.85 ± 3.9	0.11 ± 0.06	0.13 ± 0.05	0.40 ± 0.40	1.43 ± 0.53
**Non-Industrialized (305) §§**					
**Children with ASD** (n = 201)	15.88 ± 7.5	0.19 ± 0.07	0.17 ± 0.06	0.44 ± 0.42	1.72 ± 0.60
**T**ypically developing controls (n = 104)	9.53 ± 3.8	0.11 ± 0.12	0.12 ± 0.04	0.39 ± 0.38	1.36 ± 0.50
Two-way ANOVA (Group effect)	F = 68.2 *p* < 0.001**	F = 128.9 ***p*** < 0.001**	F = 5.2, *p* = 0.024*	F = 4.7, *p* = 0.031*	F = 43.8, *p* < 0.001**
Two-way ANOVA (Governorate effect)	F = 13.7 *p* < 0.001**	F = 25.3, *p* < 0.001**	F = 6.8 *p* = 0.010*	F = 9.1, *p* = 0.003**	F = 15.1, *p* < 0.001**
Two-way ANOVA Interaction Effect (Group and Governorate)	F = 7.8 ***p* = *0.006***	F = 1.8, > 0.05	F = 1.2 > 0.05	F = 1.6, > 0.05	F = 3.9 ***p* = *0.048***

§Industrialized = Cairo, Dakahlia, Damietta, Gharbia; §§ Non-Industrialized = Fayoum, Assiut, Aswan, Marsa Matrouh, *significant at p value < 0.05, **highly significant at *p* value < 0.01

**Fig. 2:**
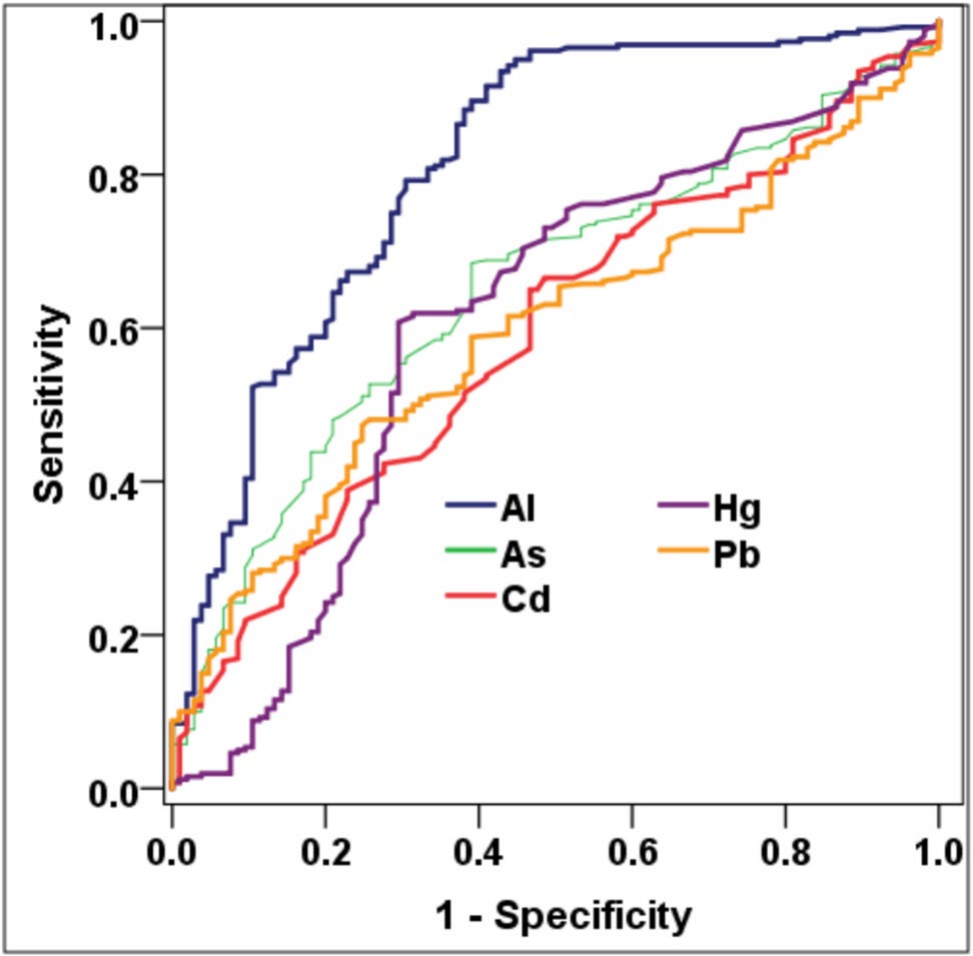
ROC curve for toxic metals in diagnosing ASD

To assess the diagnostic utility of individual toxic metals in identifying children with ASD, ROC curve analysis was performed for each of the five metals (Fig. 2). Al emerged as the most promising marker*,* demonstrating a high area under the ROC curve (AUC = 0.85), with a sensitivity of 89.4% at a cutoff value of ≥ 10.35 ppm*.* However, its specificity was relatively modest (61.7%*), i*ndicating that while elevated Al levels are common among children with ASD, false positives may occur when used as a sole diagnostic marker (Table 4).

**Table 4:** Diagnostic performance metrics of toxic metals for identifying ASD

Characteristics	Al	As	Cd	Hg	Pb
AUC	0.812 (0.761–0.863)	0.653 (0.594–0.712)	0.591 (0.529–0.653)	0.611 (0.544–0.679)	0.596 (0.536–0.656)
p-value	< 0.001*	< 0.001*	0.007*	0.001*	0.004*
Cut point	≥ 10.35 ppm	≥ 0.10 ppm	≥ 0.10 ppm	≥ 0.30 ppm	≥ 1.87 ppm
Sensitivity	89.4% (85.0%–92.8%)	69.6% (63.6%–75.1%)	66.9% (60.9%–72.6%)	62.4% (56.2%–68.2%)	47.5% (41.4%–53.8%)
Specificity	61.0% (50.9%–70.3%)	58.1% (48.1%–67.7%)	45.7% (36.0%–55.7%)	68.6% (58.8%–77.3%)	74.3% (64.8%–82.3%)
DA	81.0% (76.7%–84.9%)	66.1% (61.0%–70.9%)	61.0% (55.8%–66.0%)	62.9% (57.7%–67.8%)	55.0% (49.8%–60.2%)
YI	**50.0% (39.9%–60.0%)**	27.4% (16.5%–38.4%)	12.8% (1.7%–23.8%)	30.3% (19.8%–40.9%)	21.6% (11.3%–31.9%)
PPV	85.1% (80.4%–89.1%)	80.6% (74.9%–85.5%)	75.6% (69.6%–81.0%)	83.6% (77.5%–88.6%)	82.2% (75.2%–88.0%)
NPV	**68.8% (58.4%–78.0%)**	43.0% (34.7%–51.5%)	35.6% (27.5%–44.2%)	41.1% (33.8%–48.7%)	35.9% (29.6%–42.7%)
LR +	2.28 (1.79–2.91)	1.65 (1.30–2.10)	1.24 (1.02–1.50)	2.03 (1.48–2.77)	1.84 (1.30–2.61)
LR-	0.18 (0.12–0.26)	0.53 (0.41–0.67)	0.72 (0.55–0.94)	0.57 (0.47–0.69)	0.71 (0.60–0.83)
LR	12.65 (7.30–21.92)	3.13 (1.96–5.00)	1.71 (1.08–2.72)	3.56 (2.19–5.79)	2.60 (1.58–4.28)

Data presented as values and 95% confidence interval. AUC: Area under curve. DA: Diagnostic accuracy. YI: Youden’s index. PPV: Positive Predictive value. NPV: Negative Predictive value. LR+: Positive likelihood ratio. LR-: Negative likelihood ratio. LR: Diagnostic odd ratio. *Significant

As and Cd also showed moderate discriminative ability*,* with AUC values of 0.78 and 0.74, respectively (Fig. 1). At optimal thresholds (As ≥ 0.10 ppm; Cd ≥ 0.10 ppm), both metals yielded acceptable sensitivity values (81.1% and 76.9%, respectively) but lower specificities (58.3% and 54.8%, respectively), limiting their clinical reliability when used in isolation. Pb exhibited moderate diagnostic utility with an AUC of 0.70, while Hg showed the lowest discriminative power (AUC = 0.62), supporting its exclusion from standalone diagnostic panels.

### Association between hair metal levels and ASD severity

**Fig. 3:**
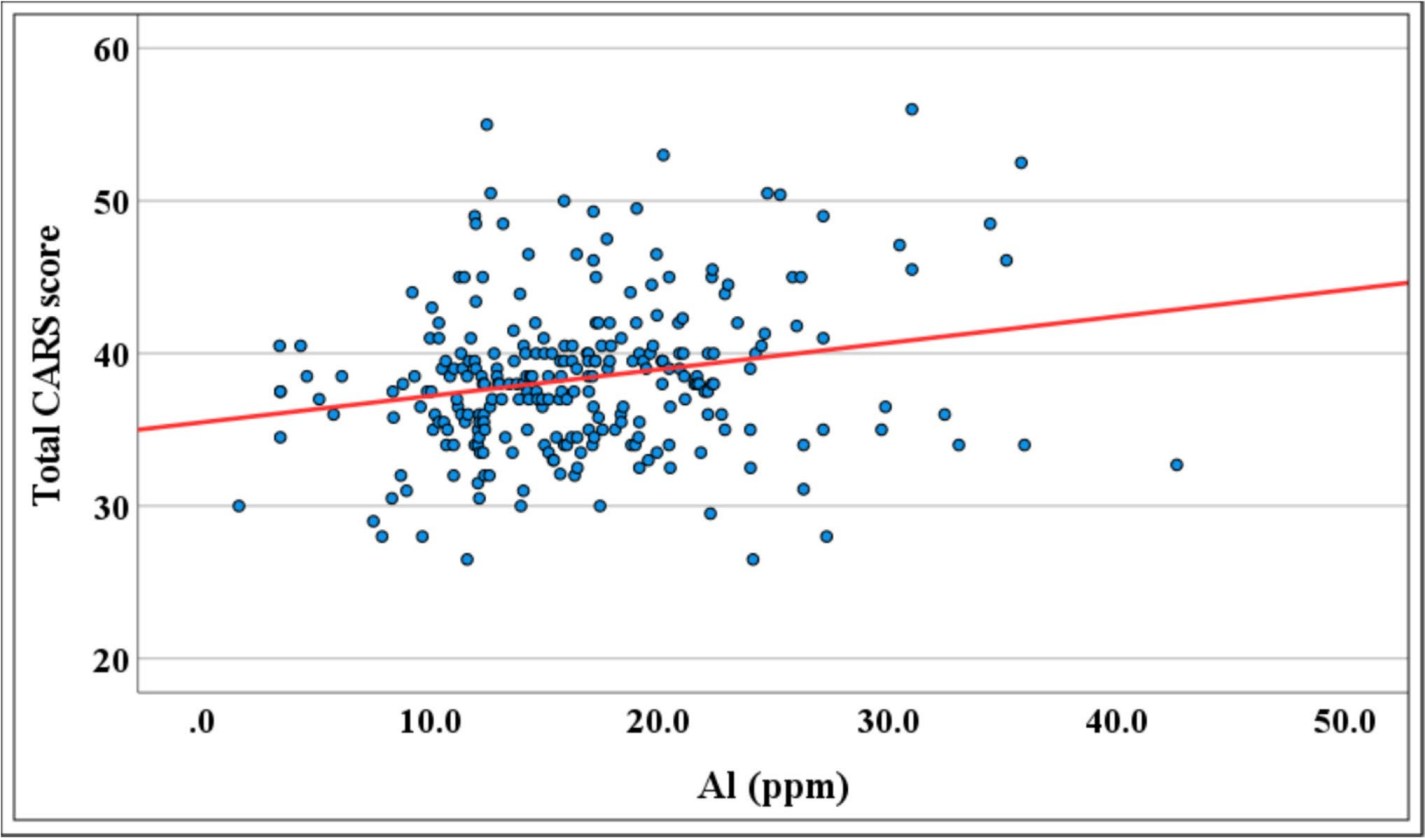
Correlation between hair Al concentrations and ASD severity (CARS scores)

Correlational analyses were conducted to examine the relationship between hair metal concentrations and ASD severity, as measured by CARS (Table 5, Fig. 3). Among all tested metals, only Al showed a statistically significant positive correlation with ASD severity scores (r = 0.43, *p* < 0.001). In contrast, no statistically significant correlations were observed for As, Cd, Pb, or Hg.

**Table 5:** Mean hair concentrations of toxic metals by ASD severity (CARS classification score)

**Toxic metals levels (ppm)**	**Total CARS score**		**Severe (Total = 257)** Mean ± SD	**Moderate (Total = 120)** Mean ± SD	**Mild** **(n = 78)** Mean ± SD	^ **p-value**
	**r§**	**p-value#**				
Al	0.22	< 0.001*	16.76 ± 5.91	16.25 ± 6.76	15.77 ± 8.47	0.782
As	−0.03	0.609	0.22 ± 0.43	0.26 ± 0.44	0.22 ± 0.30	0.713
Cd	0.10	0.103	0.18 ± 0.20	0.17 ± 0.14	0.21 ± 0.11	0.674
Hg	−0.08	0.200	0.44 ± 0.35	0.50 ± 0.52	0.73 ± 0.53	0.169
Pb	0.22	< 0.001*	1.83 ± 1.18	1.66 ± 1.02	2.33 ± 0.98	0.205

§ r = Pearson correlation test. #p-value for r, ^ p-values are based on one way ANOVA between means, **highly significant at p value < 0.01.

Across mild, moderate, and severe ASD subgroups, mean Al, As, Cd and Pb concentrations increased slightly with severity, though none reached statistical significance (*p* > 0.05). (Table 5

### Distribution of autism severity across Al exposure quartiles

We re-analyzed the data using aluminum quartiles (Table 6) to examine the dose–response trend between aluminum exposure and ASD severity. Children with ASD were classified into four quartiles based on hair-Al concentration (Q1 ≤ 12.15 ppm; Q2 = 12.16–15.54 ppm; Q3 = 15.55–20.14 ppm; Q4 > 20.14 ppm). The proportion of severe ASD cases increased progressively from 20.6% in Q1 to 26.8% in Q4, while mild cases were most frequent in Q1 (57.7%). Although the trend did not reach conventional statistical significance (*p* = 0.071, Fisher’s Exact), it indicates a dose-dependent pattern consistent with the observed positive correlation (r = 0.22).

**Table 6:** Distribution of autism severity across Aluminum exposure quartiles among children with ASD

Aluminum Quartile	Severe (n = 257)	Moderate (n = 120)	Mild (n = 78)	Total (n = 455)
First (≤ 12.15)	53 (20.6%)	36 (30.0%)	45 (57.7%)	115 (25.3%)
Second (> 12.15- ≤ 15.86)	64 (24.9%)	29 (24.2%)	0 (0.0%)	116 (25.5%)
Third (> 15.86- ≤ 20.14)	71 (27.7%)	31 (25.8%)	0 (0.0%)	112 (24.6%)
Fourth (> 20.14)	69 (26.8%)	24 (20.0%)	33 (42.3%)	112 (24.6%)
*P value#*	0.071			

#Test of significant was: Fissure exact test.

## Discussion

Research into reliable biomarkers for neurodevelopmental disorders, particularly ASD, remains limited despite increasing demand [20, 43, 44]. In Egypt, recent national estimates indicate a prevalence of 1.1% among children aged 1 to 12 years [5]. This notable rise has intensified scientific efforts to explore potential environmental contributors to ASD, including the role of toxic metal exposures.

In this case–control study, we examined hair concentrations of five toxic metals; Al, Pb, Hg, As, and Cd in children diagnosed with ASD and matched typically developing relatives. Consistent with global trends, the ASD group was predominantly male (80.9%), aligning with the widely reported male-to-female ratio of 4:1 [45, 46].Theories such as altered fetal testosterone exposure may offer partial explanations for this gender disparity [46, 47].

### Aluminum: Key Marker

Children with ASD had significantly higher hair levels of Al compared to controls. The mean concentration in ASD children was 16.55 ± 6.26 ppm, significantly exceeding that in typically developing controls (9.94 ± 5.04 ppm, p < 0.001). A positive correlation was observed between Al levels and ASD severity. This pattern is consistent with earlier Egyptian studies reporting elevated Al levels in children with ASD [10, 16]. Comparable findings were also reported in neighboring Jordan, where Rashaid et al. (2021) observed significantly increased Al and trace element concentrations in scalp hair samples of children with severe ASD, and Al-Momani et al. (2019) found elevated elemental levels in blood and urine among Jordanian ASD cohorts, reinforcing the regional evidence linking Al exposure with autism-related neurotoxicity[37, 48]

The observed dose–response trend between Al burden and ASD severity reinforces the hypothesis that cumulative exposure to toxic metals contributes to neurobehavioral dysfunction through progressive neurotoxic mechanisms. This interpretation is consistent with meta-analytic evidence demonstrating elevated concentrations of Al, Pb, and Hg among children with ASD, particularly in developing countries where environmental regulation is less stringent [19]. When Al concentrations were examined by quartiles, a progressive increase in the proportion of severe ASD cases was observed, supporting a subtle but biologically plausible dose–response pattern. The subtle but consistent gradient detected in our cohort underscores that even moderate elevations in Al may exacerbate oxidative stress, impair antioxidant defenses, and potentiate neuroinflammatory cascades implicated in ASD symptom progression [49, 50]. These findings collectively highlight the need to conceptualize toxic metal exposure as a continuous risk factor rather than a binary determinant, and to consider country-specific environmental and industrial contexts when interpreting metal–neurodevelopment associations. [49, 50].

### Lead: Confirmatory but Limited

The mean Pb level was 1.79 ± 1.13 ppm in children with ASD versus 1.44 ± 0.74 ppm in typically developing controls (*p *< 0.001). This observation supports earlier studies highlighting Pb exposure in ASD pathogenesis [12]. Proximity to industrial zones or lead-containing materials may partly explain this increase. Nonetheless, its diagnostic sensitivity (47.5%) and specificity (74.3%) suggest that Pb is not a definitive biomarker.

### Cadmium and Arsenic: Sensitive but Not Specific

Children with ASD exhibited higher Cd concentrations (0.18 ± 0.18 ppm) compared to typically developing controls (0.13 ± 0.11 ppm, *p* = 0.002). Although some reports have shown conflicting results [51, 52], our findings align with those suggesting impaired detoxification pathways in ASD children may facilitate Cd accumulation [44, 53, 54].

Similarly, the ASD group had significantly elevated As levels (0.23 ± 0.43 ppm) compared to typically developing controls (0.11 ± 0.10 ppm, *p* < 0.001), corroborating findings from prior studies [53, 55].

### Mercury: Inconclusive Marker

Although Hg levels were slightly higher in ASD children (0.47 ± 0.43 ppm) than in typically developing controls (0.39 ± 0.40 ppm), the difference was not statistically significant. These results align with some studies in Egypt and Saudi Arabia [45, 53], and are consistent with findings from Jordan, where Rashaid et al. (2021) reported increased Hg and other trace element concentrations in scalp hair of children with severe ASD compared with controls [37]. However, they contrast with others reporting reduced Hg levels in children with ASD but contrast with others reporting reduced Hg levels in children with ASD [21]. Given sparse data on Hg pollution in Egypt, interpretation remains challenging [56].

### Geographic variation of the studied toxic metals across governorates

In addition to overall differences between children with ASD and typically developing controls, our extended analysis revealed significant geographic variation in toxic metal concentrations across Egyptian governorates. One-way ANOVAs demonstrated that Al, As, Cd, and Pb levels were consistently higher in industrialized urban centers such as Cairo, Dakahlia, Damietta, and Gharbia compared with frontier or Upper Egypt regions (e.g., Marsa Matrouh, Aswan). This finding is biologically plausible, as industrial emissions, high-intensity agriculture, and legacy pollution in the Nile Delta and Greater Cairo have been repeatedly linked with elevated environmental metal burdens [39–41] In contrast, lower levels in frontier governorates likely reflect reduced anthropogenic sources [42]. These observations align with the broader literature identifying Al, Pb, As, and Cd as key environmental neurotoxicants implicated in developmental disorders [43, 57]. The stronger elevation of Al, As, and Pb in children with ASD across nearly all regions highlights the potential interaction between background environmental exposures and individual susceptibility in modulating neurodevelopmental risk.

### Diagnostic utility of toxic metals

Evaluating hair metal concentrations presents an opportunity to identify potential biomarkers for ASD diagnosis. In our study, we assessed diagnostic metrics such as sensitivity, specificity, and predictive values to determine the utility of each metal.

Al demonstrated the highest diagnostic potential. A threshold of > 10.35 ppm yielded 85.1% sensitivity, suggesting its value in screening. However, its low specificity and NPV (68.8%) imply that elevated Al levels are not exclusive to ASD, limiting its utility for definitive diagnosis.

Pb had moderate specificity (74.3%) and a strong PPV (82.2%) when levels exceeded 1.87 ppm, indicating its potential as a confirmatory marker. Similarly, Hg showed a PPV of 83.6% above 0.31 ppm, although variability in literature limits its standalone use.

As and Cd showed high sensitivity but lower specificity, leading to more false positives. For example, Cd had a negative likelihood ratio of 0.72, meaning its low levels only moderately reduced ASD likelihood. As followed a similar trend, suggesting limited utility as standalone diagnostic tools.

### Exclusionary potential

Low Cd levels (< 0.10 ppm) excluded ASD with 72% certainty, while low Pb levels (< 1.87 ppm) excluded ASD with 71% certainty. These metrics suggest some value in ruling out ASD when these metals are below threshold levels.

### Clinical Translation and Diagnostic Gaps

No single metal can diagnose ASD with high accuracy. Al is suitable for screening due to high sensitivity, while Pb and Hg offer stronger confirmatory value. As and Cd may add diagnostic context but are unreliable in isolation. A multimodal approach incorporating metals with clinical, genetic, and environmental factors is likely to yield better diagnostic accuracy.

Gene–environment interactions may also play a role in modulating metal accumulation and detoxification pathways. Variability in metal levels may reflect genetic polymorphisms affecting enzyme function, transport proteins, or epigenetic regulation. Future diagnostic protocols should integrate such genetic considerations alongside environmental assessments [51, 58–61]*.*

### Environmental Risks and Future Directions

The study findings highlight the need for public health measures to reduce environmental exposure, particularly to Al and Pb. Future studies should explore gene-environment interactions and use longitudinal designs to clarify causality. Multi-biomarker strategies incorporating blood or urine samples could further validate hair-based diagnostics. To establish the clinical utility of the proposed cutoff values, external validation using independent cohorts from diverse geographic, ethnic, and environmental backgrounds is essential. Such validation should include the application of standardized analytical protocols across laboratories and collaboration with multiple centers to assess generalizability. Employing blinded replication and prospective cohort designs will also be critical to confirm the reproducibility and diagnostic accuracy of these thresholds in real-world settings.

National environmental surveillance are consistent with the environmental disparities in Egypt that were reported in our study indicating the elevated toxic metal burdens in industrial zones of Greater Cairo and in agricultural soils and irrigation water in the Nile Delta [62] Such evidence supports prioritizing region-specific policies to mitigate exposure in high-burden areas while maintaining preventive measures in frontier regions.

### Strengths

Key strengths of this study include the large sample size (455 children with ASD and 230 typically developing controls) and the geographically representative, multicenter sampling across Egyptian governorates. The use of validated diagnostic instruments (GARS-2, CARS, DSM-5 criteria) and high-precision analytical techniques (ICP-QQQ) enhances the study’s internal validity. Moreover, the inclusion of five toxic metals (Al, Pb, Cd, As, Hg) allows a comprehensive assessment of metal–ASD associations across multiple exposure pathways.

### Limitations

Several limitations should be acknowledged. The cross-sectional design precludes causal inference. Genetic and metabolic factors influencing detoxification were not examined, which may partially explain inter-individual variability in metal accumulation.

Another limitation concerns the selection of age- and sex-matched relatives as controls. The use of related controls, while reducing socioeconomic bias, may have led to partial exposure overlap because of shared water sources, dietary patterns, and cooking practices. Nonetheless, the persistence of significant group differences supports an intrinsic ASD-related susceptibility to metal dysregulation. Our complementary investigation, currently under publication, further explores environmental exposure pathways; such as household metal sources, nutrition, and regional contamination to extend the interpretative framework of the current findings.

Objective quantification of environmental burden (e.g., pollution indices, distance from industrial areas) was not performed, limiting ecological correlation. Although standardized hair-washing and decontamination protocols were employed, hair analysis remains susceptible to minor external contamination and reflects regional exposure patterns that may limit generalizability beyond the Egyptian context.

## Conclusions and recommendations

Children with ASD demonstrated significantly higher hair concentrations of Al*,* Pb*,* Cd*,* and As compared to typically developing controls. Among these, Al showed a positive correlation with ASD symptom severity and the strongest diagnostic sensitivity, suggesting potential utility as *a* screening biomarker*.* However, no single metal should be used as a standalone diagnostic indicator.

These findings highlight the potential contribution of toxic metal exposure to ASD pathophysiology while underscoring the importance of context-specific interpretation. The observed associations are most relevant to the Egyptian environmental and industrial setting and should not be generalized without validation in other populations.

Future work should integrate toxicological, genetic, and environmental components through multicenter, longitudinal studies to establish causal pathways and standardized reference ranges. Harmonized analytical protocols across laboratories are essential to ensure reproducibility and comparability. Integrating validated multi-metal screening within broader pediatric risk assessment frameworks could enhance early detection of environmentally influenced neurodevelopmental risk.

## Data Availability

">All data generated or analyzed during this study are available from the corresponding author upon reasonable request. The datasets generated and/or analysed during the current study are not publicly available due to ethical restrictions involving human subjects and the inclusion of sensitive health-related information that could potentially compromise participant privacy and confidentiality. Additionally, the informed consent obtained from participants did not include provisions for public data sharing. Furthermore, parts of the dataset are being used in ongoing related analyses that are intended for future publication. However, the data are available from the corresponding author upon reasonable request and subject to institutional and ethical approvals.

## List of Abbreviations

ASD: Autism Spectrum Disorder
DSM-5: Diagnostic and Statistical Manual of Mental Disorders, Fifth Edition
GARS-2: Gilliam Autism Rating Scale-2
Al: Aluminum
As: Arsenic
Cd: Cadmium
Hg: Mercury
Pb: Lead
ICP-MS: Coupled Plasma Mass Spectrometry
ROC: Receiver Operating Characteristic
AUC: Area Under The Curve
VABSA: Arabic version of Vineland Adaptive Behavior Scales
M-CHAT: Modified Checklist for Autism in Toddlers
CARS: Childhood Autism Rating Scale
CAPMAS: Central Agency for Public Mobilization and Statistics
ICP-QQQ, Agilent 8800: Inductively Coupled Plasma Triple Quadrupole Mass Spectrometry
OR: Odds Ratios
CI: Confidence Intervals
MOHP: Ministry of Health and Population
